# A new diagnostic method for chronic obstructive pulmonary disease using the photoplethysmography signal and hybrid artificial intelligence

**DOI:** 10.7717/peerj-cs.1188

**Published:** 2022-12-19

**Authors:** Engin Melekoglu, Umit Kocabicak, Muhammed Kürşad Uçar, Cahit Bilgin, Mehmet Recep Bozkurt, Mehmet Cunkas

**Affiliations:** 1 Computer Engineering, Sakarya University, Sakarya, Turkey; 2 Electrical and Electronics Engineering, Sakarya University, Sakarya, Turkey; 3 Faculty of Medicine, Sakarya University, Sakarya, Turkey; 4 Electrical and Electronics Engineering, Selcuk University, Konya, Turkey

**Keywords:** Signal processing in biomedical, Photoplethysmography signal, Machine learning algorithm, Chronic obstructive pulmonary disease

## Abstract

**Background and Purpose:**

Chronic obstructive pulmonary disease (COPD), is a primary public health issue globally and in our country, which continues to increase due to poor awareness of the disease and lack of necessary preventive measures. COPD is the result of a blockage of the air sacs known as alveoli within the lungs; it is a persistent sickness that causes difficulty in breathing, cough, and shortness of breath. COPD is characterized by breathing signs and symptoms and airflow challenge because of anomalies in the airways and alveoli that occurs as the result of significant exposure to harmful particles and gases. The spirometry test (breath measurement test), used for diagnosing COPD, is creating difficulties in reaching hospitals, especially in patients with disabilities or advanced disease and in children. To facilitate the diagnostic treatment and prevent these problems, it is far evaluated that using photoplethysmography (PPG) signal in the diagnosis of COPD disease would be beneficial in order to simplify and speed up the diagnosis process and make it more convenient for monitoring. A PPG signal includes numerous components, including volumetric changes in arterial blood that are related to heart activity, fluctuations in venous blood volume that modify the PPG signal, a direct current (DC) component that shows the optical properties of the tissues, and modest energy changes in the body. PPG has typically received the usage of a pulse oximeter, which illuminates the pores and skin and measures adjustments in mild absorption. PPG occurring with every heart rate is an easy signal to measure. PPG signal is modeled by machine learning to predict COPD.

**Methods:**

During the studies, the PPG signal was cleaned of noise, and a brand-new PPG signal having three low-frequency bands of the PPG was obtained. Each of the four signals extracted 25 features. An aggregate of 100 features have been extracted. Additionally, weight, height, and age were also used as characteristics. In the feature selection process, we employed the Fisher method. The intention of using this method is to improve performance.

**Results:**

This improved PPG prediction models have an accuracy rate of 0.95 performance value for all individuals. Classification algorithms used in feature selection algorithm has contributed to a performance increase.

**Conclusion:**

According to the findings, PPG-based COPD prediction models are suitable for usage in practice.

## Introduction and literature review

Chronic obstructive pulmonary disease (COPD) is characterized by breathing signs and symptoms and airflow challenges because of anomalies in the airways and the alveoli that occurs as a result of significant exposure to harmful particles and gases. COPD is a widespread, preventable, and curable disease (Zubaydi et al., 2017; Melekoğlu et al., 2021; Batum et al., 2015). COPD constitutes a significant portion of chronic respiratory diseases. COPD is one of the maximum crucial reasons for mortality and morbidity, and with every passing day, it keeps inflicting a growing sizable financial and social burden (Lopez, 2006; Arslan & Ünsar, 2021). With the expected prolongation of life expectancy and increased exposure worldwide, the burden of COPD is predicted to increase further (Arslan & Ünsar, 2021). According to an investigation carried out with the aid of using the WHO (López-Campos, Tan & Soriano, 2016), COPD is the fourth leading reason for demise worldwide. Every year, 2.9 million people worldwide die from COPD-related diseases.

COPD is a lung complaint that prevents comfortable and healthy breathing due to the narrowing of the airways. The most common symptoms of COPD, as a progressive chronic disease for which a definitive treatment has not been found yet, are cough with phlegm and shortness of breath. In this disease, which manifests itself in the form of different symptoms depending on its stages, shortness of breath, even with light effort, indicates that the disease has progressed. One of the most important features of the symptoms is cigarette smoke. Both active cigarette smokers and nonsmokers or passive smokers around them are affected.

COPD is a progressive disease that develops due to non-microbial inflammation in the airways caused by prolonged exposure to tobacco smoke, noxious gases, and particles. As a result of this inflammation, while the airways are gradually narrowing, irreversible enlargement and destruction of the air sacs (alveoli) occur in the lung tissue (Amaral et al., 2015).

Because there is insufficient information, approximately COPD, analysis, diagnosis, and treatment are delayed. The specialized doctor makes the diagnosis based on the information collected from the spirometer gadget, which is the approach utilized to diagnose the ailment. These methods are only applied in hospitals and performed by technicians. It is important to monitor the patient’s illness after diagnosis and to monitor the damage the disease has caused to the patient’s body. Early diagnosis and intervention in COPD can stop or slow the progression of the disease. COPD, or chronic obstructive pulmonary disease, is caused by the narrowing of the airways in the lungs that make breathing difficult, and because the disease is often permanent and progressive, diagnosing the disease in its early stages can leave less harm to the patient. Monitoring at regular intervals is very important in terms of the course of the disease. This process can only be performed in hospitals. It is a complicated and time-consuming process (Zubaydi et al., 2017).

The diagnosis of COPD is made by using the spirometer device. The spirometer should measure forced vital capacity (FVC), and volume exhaled (FEV1) within 1 s of this maneuver and calculated the FEV1/FVC ratio. A medical professional can make a diagnosis by comparing spirometry measurements with reference values determined by age, height, weight, and BMI. When we divide FEV1 by FVC, it is considered to be less than 70% a COPD patient (Melekoğlu et al., 2021; Isik, Guven & Buyukoglan, 2015; Uçar et al., 2018b). The difficulties of using the spirometer device can be experienced, especially in small children, the disabled, and patients with advanced illnesses. This necessitates shortening and facilitating the diagnosis time (Melekoğlu et al., 2021; Er & Temurtas, 2008; Er et al., 2009). Because of these drawbacks, there is a need to design methods that are simple to use and follow in order to diagnose COPD more effectively (Uçar et al., 2018b, 2018c). To overcome these problems, in order to make the COPD diagnoses process faster and then patient monitoring easier, it is taken into consideration that the usage of the photoplethysmography (PPG) signal can be beneficial withinside the diagnosis of COPD (Melekoğlu et al., 2021; Moraes et al., 2018). The PPG is a biological signal that may be measurable anywhere near the heart.

Heart signals convey vital information about the body and illness. Therefore, based on the obtained results, it has been evaluated that it can be used in the diagnosis of COPD. A PPG signal-based COPD diagnostic method is suggested in this study. It is expected that the developed method will also create an infrastructure for the production of portable devices for the diagnosis of the disease and be low in cost.

Studies in the literature show that artificial intelligence algorithms can be used in the detection of asthma and COPD (Joumaa et al., 2022). In the related study, the use of open source datasets is also recommended. In addition to machine learning algorithms, the use of deep learning algorithms is increasing in the diagnosis of medical diseases. The development of computer infrastructures increases deep learning applications. Deep learning has higher performance compared to classical machine learning algorithms (Ghorbanzadeh et al., 2019; Sahoo, Pradhan & Das, 2020; Zhang et al., 2017).

In recent years, in the diagnosis of diseases, various types of research areas have been carried out on the usability of some new and helpful classifiers, decision-making software, and tools (de Mesquita et al., 2022; Lazazzera et al., 2021). One of these areas is artificial intelligence applications (Valente et al., 2016; Rodrigues et al., 2018). It is clear that these systems will provide advantages such as assistance in making the diagnosis, shortening the diagnosis time, efficiency, and increased productivity, which will benefit the medical field (Filho et al., 2014). This study intends to diagnose COPD with the machine learning algorithm only by using the PPG signal belonging to a patient.

One of the overall goals of this study is to facilitate the diagnosis of COPD through machine learning, which helps to confirm the diagnosis of COPD. In addition, the improvement of parameters such as diagnosis duration, efficiency, and time are among the objectives (Isik, Guven & Buyukoglan, 2015). This study was carried out using the PPG signal in compliance with the principles of the GOLD (Global Initiative for Chronic Obstructive Lung Disease).

The aim of this study is to diagnose COPD quickly and reliably with artificial intelligence-based PPG signal. In this study, a different and improved model from the literature is proposed. PPG records were collected from patients and healthy individuals for model formation. PPG signals are noise-free and split into sub-frequency bands. Then, features in the time domain are extracted from each frequency band. Feature selection algorithm is used to improve performance and eliminate unnecessary features. With the obtained feature sets, classification was made with the help of machine learning algorithms. The results showed that the diagnosis can be made with a two-second PPG signal.

## Method and material

**Figure 1:**
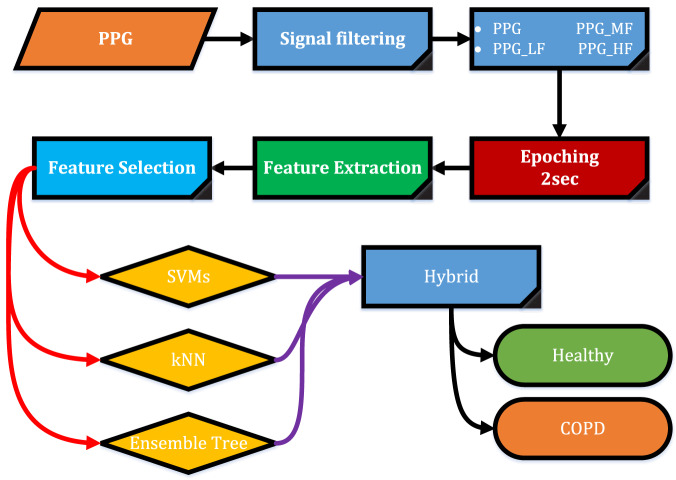
Diagram flow.

To explain the purpose of the study, the diagram in Fig. 1 was followed. Firstly, PPG is separated into sub-frequency bands with the help of digital filters. It is then split into two-second epochs. Time domain features were extracted from each epoch. In order to increase performance, the best features are selected with the help of a feature selection algorithm. Selected features are classified by the Ensemble Tree algorithms (ET), k-nearest neighbor algorithm (kNN), support vector machines (SVMs), and hybrid methods.

### Data collection

The data used in the study were obtained from the Sleep Laboratory of Sakarya Hendek State Hospital. The data in question; has been examined and diagnosed by a medical professional according to the criteria for COPD and is classified as either diseased or healthy. In order to carry out the research, permissions were obtained from the ethics committee report numbered 1614662/050.01.04/70 from the Dean of the Faculty of Medicine University from Sakarya and R.T Ministry of Health Republic of Turkey, Turkey Public Hospitals Institution Sakarya Province Public Hospitals Association General Data Secretary, and usage permission numbered 94556916/904/151.5815. A consent form was obtained from all participants. The data used in the study were collected in 2015–2016.

Within the scope of the study, the studies have been made on identified patients, six healthy and eight patient, 12 of them male and two female, fourteen people in total. Personal demographic records and COPD registry records are given withinside Table 1.

**Table 1:** Distribution of demographic information and records about individuals.

	Female			Male			All individuals		
	n1 = 2			n2 = 12			n = n1 + n2 = 14		
	Mean		SD	Mean		SD	Mean		SD
Age (year)	55.50	$\pm$	4.95	53.17	$\pm$	9.43	53.50	$\pm$	8.82
Weight (kg)	105.50	$\pm$	6.36	101.92	$\pm$	8.08	102.43	$\pm$	7.75
Height (cm)	170.00	$\pm$	7.07	173.42	$\pm$	6.52	172.93	$\pm$	6.43
BMI (kg/m2)	36.70	$\pm$	5.23	33.75	$\pm$	2.54	34.17	$\pm$	2.96
Photoplethysmography time distribution record (Sec)									
	Mean		SD	Mean		SD	Mean		SD
COPD group	–	$\pm$	–	28,643.50	$\pm$	11,082.52	2,8643.50	$\pm$	11,082.52
Control group	2,6041.00	$\pm$	4,963.89	32,611.00	$\pm$	5,351.56	3,0421.00	$\pm$	5,798.47

**Note**:

BMI, Body Mass Index.

### Signal pre-processing

A digital filter is applied to the PPG signal, and a new PPG signal is obtained, which has a sub-frequency band PPG signal. In an attempt to eliminate noise from the PPG signal, a Chebyshev type II bandpass filter with frequencies ranging from 0.1 to 20 Hz was used, followed by a “Moving Average” filter, and the PPG signal was received without noise (Şahan et al., 2007). During the study, three sub-frequency bands for the PPG signal were acquired throughout the investigation. These are sub-frequency (LF) band range of 0.04 to 0.15 Hz, (MF) mid-frequence (MF) band range of 0.09 to 0.15 Hz, and high-frequency (HF) band range of 0.15 to 6 Hz (Uçar et al., 2018a). At the end of the filtering operations, the obtained signals (PPG, $PP{G_{LF}}$, $PP{G_{MF}}$, $PP{G_{HF}}$) were split into $T = 2$ s epochs, and 25 features were obtained from the time domain of each epoch. Obtained epoch information is shown in Table 2.

**Table 2:** Epoch distribution.

	No	Gender	Epoch count
COPD	1	F	14,323
	2	F	22,213
	3	M	2,248
	4	M	16,228
	5	M	13,978
	6	M	15,673
	7	M	14,428
	8	M	13,093
	Total		112,184
Healthy	9	M	17,263
	10	M	19,393
	11	M	15,463
	12	M	15,463
	13	M	15,463
	14	M	14,773
	Total		97,818

**Figure 2:**
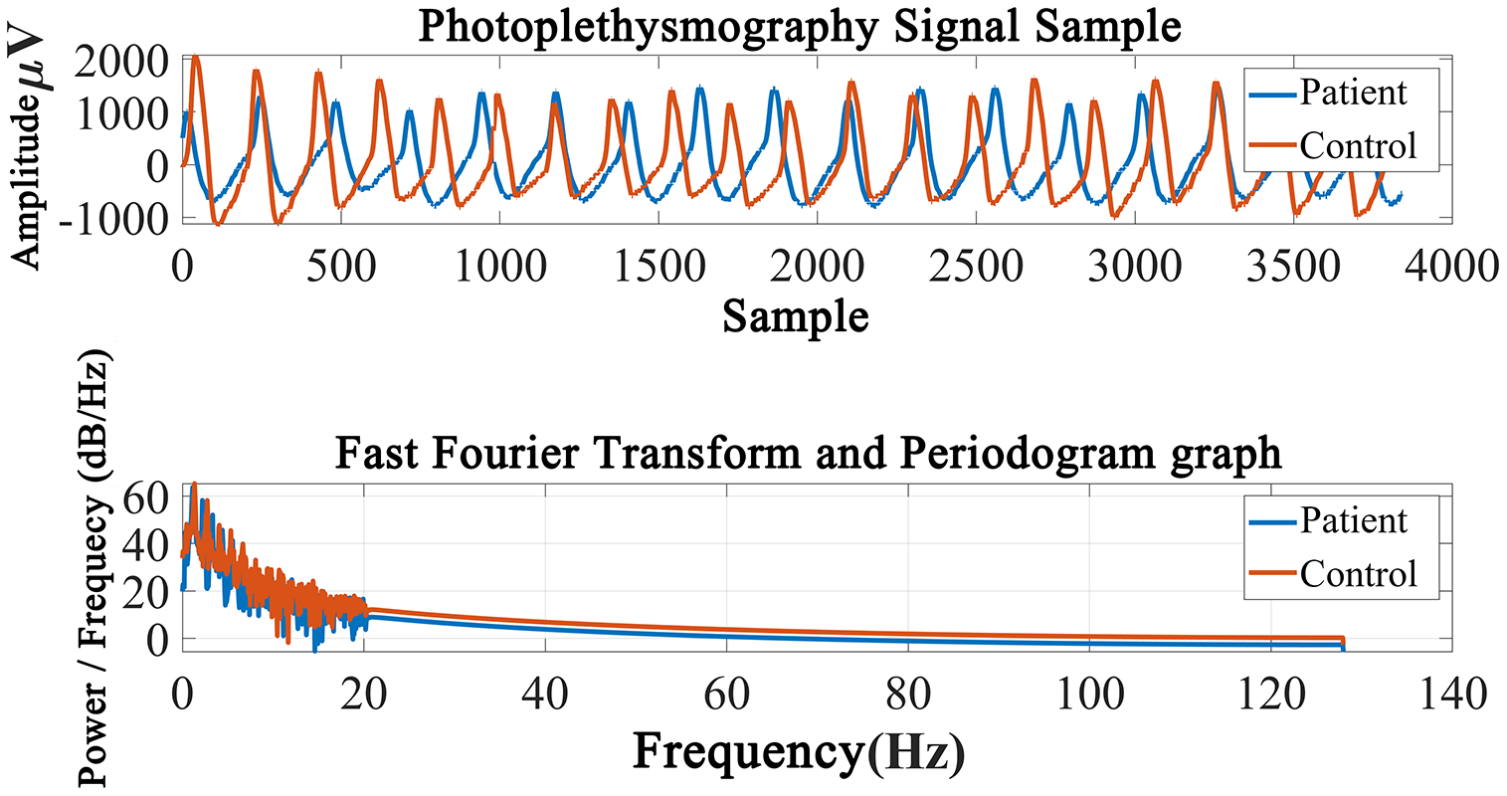
Periodogram graph of the photoplethysmography signal.

Figure 2 shows the PPG record of the COPD and Control groups and the periodogram with a Fast Fourier Transform graphic. As can be visible withinside the figure, there are variations among the sign amplitudes. Graph is a performance indicator used to show visual difference.

### Feature extraction

Four signals were obtained in the preceding process. Each of the four signals has 25 features extracted. Many features have been used for PPG signals in the literature (Uçar et al., 2021; Uçar et al., 2017). In our study, we retrieved 25 characteristics from the PPG signal. The 25 extracted features are shown in Table 3. The first three columns contain the properties number, property name, and formula information. The *x* shown in formulas represents the signal. These operations are computed using the MATLAB library (Uçar et al., 2021; Wallisch et al., 2009). An aggregate of 100 features have been extracted.

**Table 3:** Photoplethysmography properties.

No	Features name	The formula [b]
1	Kurtosis	${x_{kur}} = \displaystyle{{\sum\limits_{i = 1}^n {(x(} i) - \bar x{)^4}} \over {(n - 1){S^4}}}$
2	Skewness	${x_{ske}} = \displaystyle{{\sum\limits_{i = 1}^n {{{({x_i} - \bar x)}^3}} } \over {(n - 1){S^3}}}$
3	*Interquartile width	$IQR = iqr(x)$
4	Coefficient of variation	$DK = (S/\bar x)100$
5	Geometric average	$G = \root n \of {{x_1} \times \cdots \times {x_n}}$
6	Harmonic average	$H = n/\bigg(\displaystyle{1 \over {{x_1}}} + \cdots + \displaystyle{1 \over {{x_n}}}\bigg)$
7	Hjort activity coefficient	$A = {S^2}$
8	Hjort mobility coefficient	$M = S_1^2/{S^2}$
9	Hjort complexity coefficient	$C = \sqrt {{{(S_2^2/S_1^2)}^2} - {{(S_1^2/{S^2})}^2}}$
10	*Maximum	${x_{max}} = max({x_i})$
11	Median	$\bar x\, = \,\left\{ \matrix{{x_{\displaystyle{{n + 1} \over 2}}}{\rm }\,\,\,\,\,\,\,\,\,\,\,\,\,\,\,\,\,\,{\rm : }\;x\;{\rm tek} \cr \displaystyle{1 \over 2}\bigg({x_{\displaystyle{n \over 2}}} + {x_{\displaystyle{n \over 2} + 1}}\bigg){\rm : }\;x\;{\rm ift} } \right.$
12	*Median absolute deviation	$MAD = mad(x)$
13	*Minimum	${x_{min}} = min({x_i})$
14	*Moment, central moment	$CM = moment(x,10)$
15	Average	$\bar x = \displaystyle{1 \over n}\sum\limits_{i = 1}^n = \displaystyle{1 \over n}({x_1} + \cdots + {x_n})$
16	Average curve length	$CL = \displaystyle{1 \over n}\sum\limits_{i = 2}^n | {x_i} - {x_{i - 1}}|$
17	Average energy	$E = \displaystyle{1 \over n}\sum\limits_{i = 1}^n {x_i^2}$
18	Average square root RMS value	${X_{rms}} = \sqrt {\displaystyle{1 \over n}\sum\limits_{i = 1}^n | {x_i}{|^2}}$
19	Standard error	${S_{\bar x}} = S/\sqrt n$
20	Standard deviation	$S = \sqrt {\displaystyle{1 \over {n - 1}}\sum\nolimits_{i = 1}^n {{{({x_i} - \bar x)}^2}} }$
21	Shape factor	$SF = {X_{rms}}/\bigg(\displaystyle{1 \over n}\sum\nolimits_{i = 1}^n {\sqrt {|{x_i}|} } \bigg)$
22	*Singular value decomposition	$SVD = svd(x)$
23	*25% Trimmed mean value	$T25 = trimmean(x,25)$
24	*50% Trimmed mean value	$T50 = trimmean(x,50)$
25	Average teager energy	$TE = \displaystyle{1 \over n}\sum\limits_{i = 3}^n {(x_{i - 1}^2 - {x_i}{x_{i - 2}})}$

**Notes:**

* The property was computed using MATLAB.

IQR, Interquartile Range; CV, Coefficient of Variation.

${S^2}$, variance of the signal *x*. *S2*

$S_1^2$, Variance of the 1st derivative of the signal *x*.

$S_2^2$, Variance of the 2nd derivative of the signal *x*.

After 25 features were extracted from each signal as indicated in the diagram flow, by using kNN, SVMs, and Ensemble Tree classification algorithm, the operations were performed on MATLAB. Additionally, by combining the algorithm, a hybrid machine learning algorithm was created.

Statistical features help to describe samples better. While a single feature does not make sense, more than one feature can become meaningful with artificial intelligence methods. Here, it is aimed to use more than one statistical parameter with artificial intelligence that has been tried before. These features have been preferred because they have been used in different studies in the literature and are efficient.

### Feature selection

The number of features in a feature selection method has an impact on machine learning performance in both positive and negative ways (Uçar et al., 2020). Negative impacts are isolated through feature selection. According to the tag prediction of any feature, the feature selection procedure ranks from relevant to irrelevant. The researcher can add as many features to the dataset as he wants, ranking them from most relevant to least relevant. As a consequence, he may receive more detailed findings and run faster program cycles without unnecessary data usage. Object selection methods are often used to select a smaller subset of more distinct objects, and in this way, the goal is to improve classification performance (Kohavi & John, 1997; Isabelle & Elisseeff, 2000; Eskidere, 2012). In this research, Fisher’s feature selection algorithm was used due to its high performance (Uçar et al., 2020). The features selected in the study are summarized in Table 4. The table shows the features’ correlation level (R), and F displays feature numbers. R indicates the level of association of attributes with the tag. F represents the feature number. The features in the table are ranked with the features with the best correlation at the top.

**Table 4:** Feature selection from signals for the entire data set.

S	PPG		PPG LF		PPG MF		PPG HF	
No	F	R	F	R	F	R	F	R
1	17	0.082	2	0.027	2	0.029	8	0.081
2	8	0.062	1	0.021	1	0.022	1	0.060
3	25	0.041	11	0.021	6	0.022	25	0.042
4	11	0.039	4	0.021	11	0.022	14	0.042
5	14	0.039	8	0.019	8	0.020	7	0.042
6	22	0.039	16	0.019	25	0.020	3	0.041
7	2	0.039	6	0.018	18	0.019	24	0.040
8	9	0.038	25	0.018	20	0.018	9	0.039
9	3	0.033	22	0.018	14	0.018	18	0.039
10	19	0.032	17	0.003	17	0.007	19	0.037
11	1	0.017	13	0.003	13	0.007	11	0.021
12	21	0.007	15	0.002	15	0.002	4	0.005
13	16	0.005	14	0.002	4	0.002	13	0.005
14	6	0.003	20	0.001	3	0.001	21	0.003
15	4	0.003	3	0.000	16	0.000	6	0.002
16	7	0.001	12	0.000	7	0.000	20	0.002
17	12	0.001	7	0.000	22	0.000	17	0.002
18	13	0.001	21	0.000	12	0.000	12	0.001
19	15	0.001	19	0.000	21	0.000	15	0.001
20	10	0.000	5	0.000	5	0.000	5	0.001
21	20	0.000	18	0.000	19	0.000	16	0.001
22	23	0.000	10	0.000	10	0.000	2	0.000
23	5	0.000	23	0.000	23	0.000	10	0.000
24	18	0.000	24	0.000	24	0.000	22	0.000
25	24	0.000	9	0.000	9	0.000	23	0.000

**Note:**

S, Signal; F, Feature; R, Correlation coefficient.

Correlation coefficients range from 0 to 1.1 indicates the highest correlation. The correlation ranges are expressed as: These are $0 < R < 0.19$—the relationship is negligible, $0.2 < R < 0.39$ weak relationship, $0.4 < R < 0.69$ moderate relationship, $0.7 < R < 0.89$ strong relationship, and $0.9 < R < 1$ is a very strong relationship.

### Machine learning

Machine learning is the modeling of systems with computers that make predictions by making inferences from operations on data by using mathematics and statistics (Arslankaya & Toprak, 2021). One of the problems that can be solved by machine learning is the classification of problems with a wide range area of uses. Today many problems can be somehow considered and solved as a classification problems. kNN, SVMs, and ET models were employed in this work. The reasons for choosing these methods are the short training duration, and the high accuracy rates (Rasool et al., 2019; Uçar et al., 2017).

During the analysis performed in order to avoid errors, a hybrid machine learning algorithm structure was created (Aydilek & Arslan, 2013, 2012; Tosunoğlu et al., 2021). Due to the fact that these methods have attained successful results in the literature, they are the most frequently used machine learning algorithms. In addition, these algorithms are suitable for transferring to embedded systems (Roscher et al., 2020; Santos, Moreno & Estombelo-Montesco, 2019; Saguil & Azim, 2019). From the data used for the training of the models, 50% was used during training and 50% during the testing phase.

Hyperparameter optimization has been made for all machine learning algorithms used. Parameters were not changed manually. All parameters are made automatically by Matlab to reduce 5-fold cross-validation loss.

#### Support vector machines algorithm (SVMs)

SVMs are among the best-supervised learning algorithms. Proposed by Cortes & Vapnik (1995) and Uçar (2017). It is predominantly based on the principle of establishing the maximum distance between the examples defined as support vectors of the decision surface of two linearly separable classes and determining the class boundaries. The maximization of the distance is written as a quadratically constrained optimization problem and converted to a dual form. Developed for linear problems, this approach can be generalized for nonlinear parsing problems using kernel transformations (Akben, Subasi & Kiymik, 2010; Fernandes de Mello & Antonelli Ponti, 2018).

**Figure 3:**
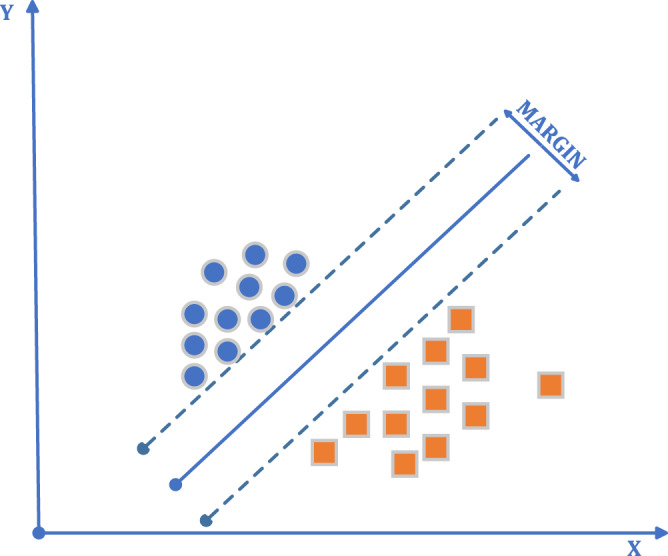
SVMs algorithm general flow diagram.

In the selection of the machine learning algorithm developed for the solution of classification problems, one of the essential criteria to be considered is the generalization performance of the algorithm (Ayhan & Erdoğmuş, 2014). In order to separate points placed on the plane, a line is drawn (Fig. 3). It intends to have this line at the maximum distance for the points of both classes. To draw the border, two lines close and parallel to each other are drawn, and these lines are brought closer together to produce a boundary line. The SVMs method is based on estimating the most appropriate function to separate the data from each other. SVMs have a simple structure and high performance in terms of practical applications, and it is pretty user-friendly. The number of samples to be used in SVMs is not essential. During training, SVMs also classify unseen data without problems. This demonstrates the generalization ability of the SVMs. The generalization feature makes the SVMs a good alternative compared to the other techniques (Kecman, 2002).

During the studies, for all classification processors, the features were divided into 10 different feature sets (5%, 10%, 15%, 20%, 25%, 30%, 35%, 40%, 45%, 50%). While applying the SVMs, the parameter optimization method was used. Using this method improves performance.

#### k-nearest neighborhood algorithm (KNN)

kNN is a basic machine-learning method that uses the supervised learning approach (Şahan et al., 2007; Uçar, 2017). Although it is used in solving both classification and regression problems, it is mostly used in solving classification problems in the industry.

kNN algorithms were proposed in 1967 by Cover & Hart (1967). The algorithm is used by utilizing the data in a sample set with certain classes. The new data that will be added to the sample data set, in accordance with the available data, the distance is calculated, and k number of close neighbors are checked. Three types of distance functions are generally used for distance calculations/these are (1) “Euclidean” Distance, (2) “Manhattan” Distance, (3) “Minkowski” Distance.

**Figure 4:**
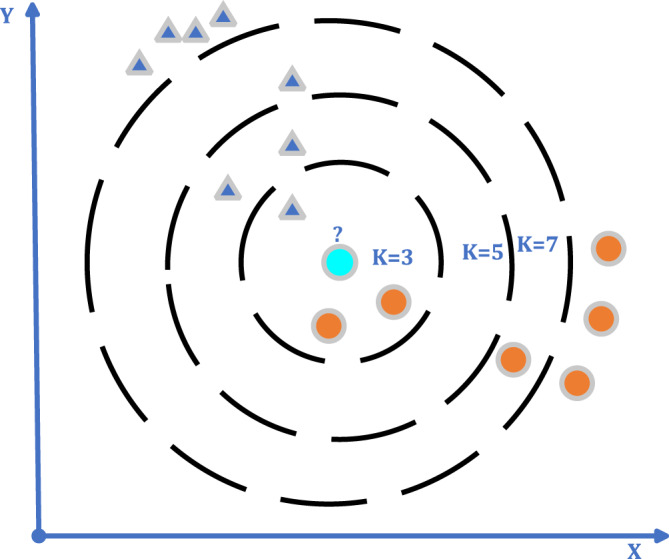
kNN algorithm general flow diagram.

The kNN algorithm may be used for both regression and classification; however, it is more commonly utilized for classification tasks (Şahan et al., 2007). By calculating the similarity of the data to be classified to the standard behavior data in the learning set; classes are assigned to the classes according to the threshold value determined by the mean of the k data, which is thought to be the closest (Fig. 4) (Duman et al., 2021). The important thing is that the characteristics of each class are clearly defined in advance. The performance of the method criteria is affected by the number of neighbors closest to k, the threshold value, the similarity measurement, and the sufficient number of expected behaviors in the learning set. Initially, for classification with kNN, the k value is selected. A large selection of k may result in the grouping of dissimilar data sets. In studies, the k value is generally preferred as 3, 5, or 7 (Uçar, 2017; Khan, Ding & Perrizo, 2002).

#### Ensemble Tree (ET)

An ensemble of trees makes predictions by gathering the results from individual decision trees (DT) (Huang, Zhao & Huang, 2021). ET is a machine learning approach that is commonly utilized in regression and classification issues (Breiman, 2001, 1996). The basic working principle of Ensemble is based on the principle of performing a simple decision-making process by making any classification problem multi-stepped state (Çölkesen & Kavzoğlu, 2017). With classification algorithms, we try to predict which class an object will be included in. Many classification methods select one suitable problem makes the necessary optimizations, and tries to achieve high accuracy rates.

Ensemble methods; It combines the prediction results of multiple base models to produce more robust and generalizable results compared to a single model. The success of these methods is based on two criteria; the learning success of the base learner and their differences from each other. Performance can sometimes drop on models.

The Ensemble Tree classifier is a system that was constructed by merging many classification methods to give more consistent and dependable predictions. The system is built with N odd or even classifiers. During classification, the output values produced by each classifier are counted. The decision of the ensemble classifier is determined by the principle of majority vote.

In this study, three classifiers were used; ensemble classifier SVMs, kNN, and Ensemble Tree, and the study were prepared in the MATLAB environment.

#### Hybrid artificial intelligence method (HAI)

**Figure 5:**
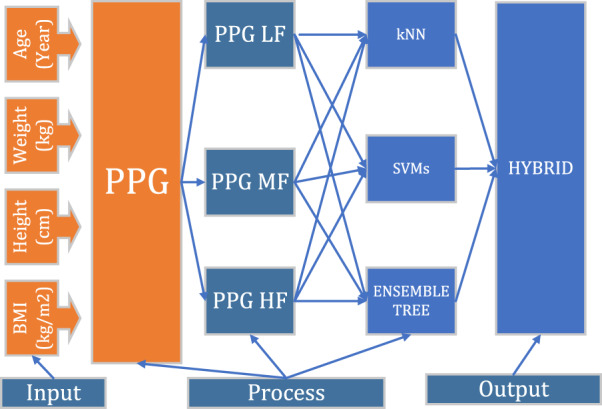
Hybrid artificial intelligence model algorithm general flow diagram.

Today, it is seen that organizations are increasingly positioning artificial intelligence instead of operational solutions and rapidly integrating it into their business processes (Deliloğlu & Çakmak Pehlivanlı, 2021). Hybrid Artificial Intelligence combines the received classification processes to produce the answer given by the majority (Fig. 5). Bringing together the weak classification to reveal the robust classification. As the number of classifiers increases, the model stability increases.

#### Performance assessment criteria

Various performance assessment criteria were utilized to examine the accuracy rates of the suggested systems. Specificity, sensitivity, kappa coefficient, accuracy rates, receiver operating characteristic (ROC), area under the ROC curve (area under a ROC—AUC), and k-fold cross-validation accuracy rate are among them (Uçar et al., 2020).

During the classification of the feature sets, they are divided into (50%) Training and (50%) Test data sets (Table 5). In the received data, 2-second epoching processes were carried out for the data obtained from 14 patients, including Healthy and Control.

**Table 5:** Training and test.

	Percent	COPD	Healthy	Total
Training	50%	56,092	53,408	109,500
Test	50%	56,092	53,407	109,499
Total	100%	112,184	106,815	218,999

From the training and test results received, the total number of sick patients is 112,184, and healthy ones are 106,815. The best performance results were acquired by using classification algorithms in the processes and combining the hybrid artificial intelligence method with classification algorithms.

For the calculation of the performance values, the confusion matrix was created, and the performance parameters were calculated (Table 6).

**Table 6:** Confusion matrix.

		Predicted	
		P	N
Actual situation	P	TP	FN
	N	FP	TN

While interpreting the Kappa value, the ranges in Table 7 are taken into account. According to these values, R values above 0.81 are very good for the system.

**Table 7:** Kappa coefficients boundary ranges.

Kappa coefficients	Explanation
0.81–1.00	Very good compatibility
0.61–0.80	Good compatibility
0.41–0.60	Moderate compliance
0.21–0.40	Low level of compliance
0.00–0.20	Poor fit
<0.00	Very poor fit

## Results

The results acquired within the scope of the study are presented in this section. The goal of this study is to use artificial intelligence with PPG signals to diagnose chronic obstructive pulmonary disease (COPD). For this purpose, the study was organized as follows: Initially, the PPG signals received from individuals (“Data collection”) are divided into three sub-frequency bands (“Signal pre-processing”). Then, 25 features are extracted to the photoplethysmography signal and in the three sub-frequency bands (“Feature extraction”). In the next step, the diagnosis of individual COPD values was estimated with the help of the feature groups feature selection algorithm (“Feature selection”). Finally, performance assessment criteria were used to evaluate the performances of the proposed models (“Performance assessment criteria”).

Within the scope of the study, COPD was estimated by using all the features of PPG and three sub-frequency bands, both separately and together in Table 8. By using PPG and all the features of the three sub-frequency bands, the estimation was made using the performance evaluation criteria in the prepared models (Table 8). It has been determined that the calculated performance evaluation criteria are very close to 1. The accuracy rate in the model created with the PPG signal is approximately 95%. It is seen that the success rates of the models belonging to the sub-frequencies of PPG are over 80%. The sensitivity and specificity values of the models are balanced and above 0.85.

**Table 8:** Results by all features for all records. Bold numbers in the table show the best results.

Signal	Model	Performance evalutaion criteria					
		Sensitivity	Specificity	Accuracy	Kappa	F-measure	AUC
PPG	kNN	0.858	0.887	87.24	0.745	0.872	0.872
	SVMs	0.922	0.950	93.59	0.872	0.936	0.936
	ET	0.939	0.967	95.31	0.906	0.953	0.954
	Hybrid	**0.940**	**0.961**	**95.10**	**0.902**	**0.951**	**0.952**
PPG LF	kNN	0.746	0.823	78.37	0.568	0.782	0.784
	SVMs	0.790	0.835	81.24	0.625	0.812	0.813
	ET	0.877	0.929	90.30	0.806	0.903	0.904
	Hybrid	**0.837**	**0.892**	**86.46**	**0.729**	**0.864**	**0.865**
PPG MF	kNN	0.768	0.846	80.64	0.613	0.805	0.807
	SVMs	0.648	0.596	62.26	0.244	0.620	0.622
	ET	0.853	0.916	88.46	0.769	0.885	0.886
	Hybrid	**0.813**	**0.877**	**84.44**	**0.689**	**0.844**	**0.854**
PPG HF	kNN	0.826	0.918	87.17	0.743	0.870	0.872
	SVMs	0.619	0.731	67.41	0.350	0.670	0.675
	ET	0.894	0.915	90.43	0.808	0.904	0.905
	Hybrid	**0.836**	**0.925**	**87.95**	**0.759**	**0.878**	**0.880**
All	kNN	0.773	0.922	84.60	0.693	0.841	0.847
	SVMs	0.641	0.839	73.79	0.478	0.727	0.740
	ET	0.928	0.951	93.96	0.879	0.939	0.940
	Hybrid	**0.828**	**0.955**	**89.03**	**0.781**	**0.887**	**0.891**

The COPD prediction model was reconstructed by reducing all feature groups with the help of the Fisher feature selection algorithm (Tables 9–13).

**Table 9:** List of PPG signals by feature selection results. Bold numbers in the table show the best results.

	Model	Performance Evalutaion Criteria					
		Sensitivity	Specificity	Accuracy	Kappa	F-measure	AUC
Level 1 5%	kNN	0.663	0.533	60.04	0.198	0.591	0.598
	SVMs	0.639	0.575	60.80	0.214	0.605	0.607
	ET	0.611	0.608	60.99	0.219	0.609	0.609
	Hybrid	**0.626**	**0.589**	**60.81**	**0.215**	**0.607**	**0.607**
Level 2 10%	kNN	0.853	0.955	90.29	0.806	0.901	0.904
	SVMs	0.854	0.907	88.03	0.760	0.880	0.881
	ET	0.841	0.901	87.01	0.741	0.869	0.870
	Hybrid	**0.853**	**0.928**	**89.02**	**0.781**	**0.889**	**0.891**
Level 3 15%	kNN	0.816	0.964	88.83	0.777	0.884	0.890
	SVMs	0.882	0.938	90.97	0.819	0.909	0.910
	ET	0.880	0.902	89.14	0.782	0.891	0.892
	Hybrid	**0.871**	**0.953**	**91.11**	**0.822**	**0.910**	**0.912**
Level 4 20%	kNN	0.850	0.963	90.57	0.811	0.903	0.907
	SVMs	0.879	0.940	90.95	0.819	0.909	0.910
	ET	0.880	0.917	89.84	0.797	0.898	0.899
	Hybrid	**0.879**	**0.959**	**91.82**	**0.837**	**0.917**	**0.919**
Level 5 25%	kNN	0.927	0.987	95.68	0.913	0.956	0.957
	SVMs	0.950	0.966	95.82	0.916	0.958	0.958
	ET	0.917	0.960	93.80	0.876	0.938	0.939
	Hybrid	**0.945**	**0.986**	**96.55**	**0.931**	**0.965**	**0.966**
Level 6 30%	kNN	0.927	0.986	95.60	0.912	0.956	0.957
	SVMs	0.941	0.968	95.43	0.908	0.954	0.955
	ET	0.932	0.969	95.06	0.901	0.950	0.951
	Hybrid	**0.951**	**0.987**	**96.90**	**0.938**	**0.969**	**0.969**
Level 7 35%	kNN	0.919	0.986	95.13	0.902	0.951	0.952
	SVMs	0.620	0.882	74.81	0.499	0.728	0.751
	ET	0.918	0.963	94.05	0.881	0.940	0.941
	Hybrid	**0.901**	**0.981**	**94.01**	**0.880**	**0.939**	**0.941**
Level 8 40%	kNN	0.911	0.983	94.66	0.893	0.946	0.947
	SVMs	0.974	0.981	97.77	0.955	0.977	0.977
	ET	0.903	0.953	92.81	0.856	0.928	0.929
	Hybrid	**0.947**	**0.987**	**96.71**	**0.934**	**0.967**	**0.968**
Level 9 45%	kNN	0.919	0.983	95.03	0.901	0.950	0.951
	SVMs	0.893	0.944	91.81	0.836	0.918	0.919
	ET	0.907	0.956	93.07	0.861	0.931	0.931
	Hybrid	**0.927**	**0.979**	**95.25**	**0.905**	**0.952**	**0.953**
Level 10 50%	kNN	0.881	0.966	92.28	0.846	0.922	0.923
	SVMs	0.922	0.935	92.87	0.857	0.929	0.929
	ET	0.890	0.942	91.55	0.831	0.915	0.916
	Hybrid	**0.922**	**0.970**	**94.60**	**0.892**	**0.946**	**0.947**

**Table 10:** PPG LF signals list by feature selection results. Bold numbers in the table show the best results.

	Model	Performance evalutaion criteria					
		Sensitivity	Specificity	Accuracy	Kappa	F-measure	AUC
Level 1 5%	kNN	0.829	0.291	56.65	0.121	0.431	0.559
	SVMs	0.936	0.184	56.98	0.123	0.308	0.561
	ET	0.948	0.170	56.89	0.121	0.288	0.559
	Hybrid	**0.943**	**0.176**	**56.94**	**0.122**	**0.297**	**0.560**
Level 2 10%	kNN	0.823	0.878	85.03	0.700	0.850	0.851
	SVMs	0.790	0.802	79.61	0.592	0.796	0.796
	ET	0.792	0.793	79.27	0.585	0.793	0.793
	Hybrid	**0.815**	**0.832**	**82.36**	**0.647**	**0.824**	**0.824**
Level 3 15%	kNN	0.925	0.965	94.50	0.890	0.945	0.945
	SVMs	0.822	0.852	83.69	0.674	0.837	0.837
	ET	0.898	0.937	91.76	0.835	0.917	0.918
	Hybrid	**0.914**	**0.959**	**93.65**	**0.873**	**0.936**	**0.937**
Level 4 20%	kNN	0.899	0.951	92.47	0.849	0.924	0.925
	SVMs	0.946	0.959	95.26	0.905	0.953	0.953
	ET	0.892	0.934	91.31	0.826	0.913	0.913
	Hybrid	**0.935**	**0.969**	**95.19**	**0.904**	**0.952**	**0.952**
Level 5 25%	kNN	0.915	0.960	93.73	0.874	0.937	0.938
	SVMs	0.608	0.680	64.34	0.288	0.642	0.644
	ET	0.898	0.941	91.96	0.839	0.919	0.920
	Hybrid	**0.890**	**0.958**	**92.36**	**0.847**	**0.923**	**0.924**
Level 6 30%	kNN	0.895	0.947	92.07	0.841	0.920	0.921
	SVMs	0.953	0.961	95.74	0.914	0.957	0.957
	ET	0.896	0.940	91.80	0.836	0.918	0.918
	Hybrid	**0.937**	**0.967**	**95.19**	**0.903**	**0.952**	**0.952**
Level 7 35%	kNN	0.884	0.947	91.52	0.830	0.915	0.916
	SVMs	0.949	0.959	95.42	0.908	0.954	0.954
	ET	0.895	0.941	91.75	0.835	0.917	0.918
	Hybrid	**0.935**	**0.968**	**95.17**	**0.903**	**0.951**	**0.952**
Level 8 40%	kNN	0.864	0.938	90.06	0.801	0.900	0.901
	SVMs	0.412	0.846	62.40	0.255	0.554	0.629
	ET	0.894	0.937	91.51	0.830	0.915	0.915
	Hybrid	**0.843**	**0.954**	**89.75**	**0.795**	**0.895**	**0.899**
Level 9 45%	kNN	0.879	0.942	91.04	0.821	0.910	0.911
	SVMs	0.933	0.950	94.14	0.883	0.941	0.941
	ET	0.893	0.937	91.48	0.829	0.914	0.915
	Hybrid	**0.928**	**0.965**	**94.61**	**0.892**	**0.946**	**0.946**
Level 10 50%	kNN	0.885	0.952	91.80	0.836	0.917	0.918
	SVMs	0.392	0.895	63.77	0.284	0.545	0.643
	ET	0.884	0.932	90.81	0.816	0.908	0.908
	Hybrid	**0.848**	**0.966**	**90.60**	**0.812**	**0.903**	**0.907**

**Table 11:** PPG MF signals list by feature selection results. Bold numbers in the table show the best results.

	Model	Performance evalutaion criteria					
		Sensitivity	Specificity	Accuracy	Kappa	F-measure	AUC
Level 1 5%	kNN	0.822	0.304	56.93	0.127	0.444	0.563
	SVMs	0.860	0.264	56.97	0.126	0.404	0.562
	ET	0.938	0.182	56.97	0.123	0.306	0.560
	Hybrid	**0.887**	**0.237**	**57.05**	**0.127**	**0.374**	**0.562**
Level 2 10%	kNN	0.916	0.965	94.04	0.881	0.940	0.941
	SVMs	0.917	0.961	93.92	0.878	0.939	0.939
	ET	0.887	0.931	90.89	0.818	0.909	0.909
	Hybrid	**0.921**	**0.969**	**94.47**	**0.889**	**0.944**	**0.945**
Level 3 15%	kNN	0.916	0.965	94.07	0.881	0.941	0.941
	SVMs	0.918	0.962	94.01	0.880	0.940	0.941
	ET	0.888	0.929	90.85	0.817	0.908	0.909
	Hybrid	**0.921**	**0.969**	**94.46**	**0.889**	**0.944**	**0.945**
Level 4 20%	kNN	0.889	0.944	91.65	0.833	0.916	0.917
	SVMs	0.957	0.975	96.58	0.931	0.966	0.966
	ET	0.901	0.937	91.92	0.838	0.919	0.919
	Hybrid	**0.935**	**0.969**	**95.17**	**0.903**	**0.951**	**0.952**
Level 5 25%	kNN	0.910	0.962	93.56	0.871	0.935	0.936
	SVMs	0.952	0.975	96.38	0.927	0.964	0.964
	ET	0.890	0.932	91.06	0.821	0.910	0.911
	Hybrid	**0.934**	**0.973**	**95.34**	**0.906**	**0.953**	**0.953**
Level 6 30%	kNN	0.911	0.964	93.67	0.873	0.936	0.937
	SVMs	0.957	0.961	95.91	0.918	0.959	0.959
	ET	0.896	0.936	91.60	0.832	0.916	0.916
	Hybrid	**0.942**	**0.974**	**95.82**	**0.916**	**0.958**	**0.959**
Level 7 35%	kNN	0.889	0.946	91.72	0.834	0.917	0.918
	SVMs	0.956	0.963	95.98	0.919	0.960	0.960
	ET	0.912	0.947	92.97	0.859	0.929	0.930
	Hybrid	**0.949**	**0.973**	**96.12**	**0.922**	**0.961**	**0.962**
Level 8 40%	kNN	0.863	0.926	89.37	0.787	0.893	0.894
	SVMs	0.942	0.952	94.73	0.894	0.947	0.947
	ET	0.911	0.946	92.85	0.857	0.928	0.929
	Hybrid	**0.937**	**0.962**	**94.96**	**0.899**	**0.949**	**0.949**
Level 9 45%	kNN	0.880	0.939	90.94	0.819	0.909	0.910
	SVMs	0.937	0.950	94.38	0.887	0.943	0.944
	ET	0.912	0.946	92.86	0.857	0.928	0.929
	Hybrid	**0.943**	**0.970**	**95.68**	**0.913**	**0.957**	**0.957**
Level 10 50%	kNN	0.881	0.939	90.96	0.819	0.909	0.910
	SVMs	0.939	0.950	94.48	0.889	0.945	0.945
	ET	0.868	0.925	89.62	0.792	0.896	0.896
	Hybrid	**0.922**	**0.963**	**94.24**	**0.884**	**0.942**	**0.943**

**Table 12:** PPG HF signals List by feature selection results. Bold numbers in the table show the best results.

	Model	Performance Evalutaion Criteria					
		Sensitivity	Specificity	Accuracy	Kappa	F-measure	AUC
Level 1 5%	kNN	0.643	0.584	61.47	0.228	0.612	0.614
	SVMs	0.647	0.577	61.36	0.225	0.610	0.612
	ET	0.665	0.561	61.46	0.227	0.608	0.613
	Hybrid	**0.658**	**0.568**	**61.48**	**0.229**	**0.610**	**0.614**
Level 2 10%	kNN	0.819	0.952	88.41	0.768	0.880	0.885
	SVMs	0.447	0.923	67.94	0.366	0.602	0.685
	ET	0.823	0.868	84.51	0.690	0.845	0.845
	Hybrid	**0.785**	**0.954**	**86.75**	**0.736**	**0.861**	**0.869**
Level 3 15%	kNN	0.893	0.968	93.02	0.860	0.929	0.931
	SVMs	0.848	0.930	88.84	0.777	0.887	0.889
	ET	0.856	0.888	87.24	0.744	0.872	0.872
	Hybrid	**0.874**	**0.948**	**91.03**	**0.821**	**0.909**	**0.911**
Level 4 20%	kNN	0.840	0.957	89.76	0.795	0.895	0.899
	SVMs	0.847	0.930	88.82	0.776	0.887	0.889
	ET	0.857	0.892	87.43	0.748	0.874	0.875
	Hybrid	**0.854**	**0.945**	**89.85**	**0.797**	**0.897**	**0.899**
Level 5 25%	kNN	0.842	0.846	84.47	0.689	0.844	0.844
	SVMs	0.881	0.937	90.89	0.818	0.908	0.909
	ET	0.868	0.898	88.30	0.766	0.883	0.883
	Hybrid	**0.887**	**0.918**	**90.24**	**0.805**	**0.903**	**0.903**
Level 6 30%	kNN	0.880	0.966	92.25	0.845	0.921	0.923
	SVMs	0.940	0.965	95.24	0.904	0.952	0.952
	ET	0.887	0.941	91.36	0.827	0.913	0.914
	Hybrid	**0.921**	**0.973**	**94.65**	**0.893**	**0.946**	**0.947**
Level 7 35%	kNN	0.911	0.978	94.42	0.888	0.943	0.945
	SVMs	0.564	0.886	72.17	0.447	0.690	0.725
	ET	0.902	0.945	92.34	0.846	0.923	0.924
	Hybrid	**0.874**	**0.977**	**92.49**	**0.850**	**0.923**	**0.926**
Level 8 40%	kNN	0.906	0.975	94.00	0.880	0.939	0.940
	SVMs	0.959	0.975	96.69	0.933	0.967	0.967
	ET	0.898	0.940	91.85	0.837	0.918	0.919
	Hybrid	**0.942**	**0.980**	**96.08**	**0.922**	**0.961**	**0.961**
Level 9 45%	kNN	0.891	0.893	89.25	0.785	0.892	0.892
	SVMs	0.867	0.922	89.40	0.788	0.893	0.894
	ET	0.889	0.941	91.48	0.829	0.914	0.915
	Hybrid	**0.913**	**0.955**	**93.35**	**0.867**	**0.933**	**0.934**
Level 10 50%	kNN	0.883	0.963	92.25	0.845	0.921	0.923
	SVMs	0.863	0.915	88.84	0.777	0.888	0.889
	ET	0.884	0.940	91.17	0.823	0.911	0.912
	Hybrid	**0.901**	**0.962**	**93.08**	**0.862**	**0.931**	**0.932**

**Table 13:** PPG all signals list by feature selection results. Bold numbers in the table show the best results.

	Model	Performance evalutaion criteria					
		Sensitivity	Specificity	Accuracy	Kappa	F-measure	AUC
Level 1 5%	kNN	0.865	0.965	91.39	0.828	0.912	0.915
	SVMs	0.868	0.922	89.45	0.789	0.894	0.895
	ET	0.860	0.888	87.40	0.748	0.874	0.874
	Hybrid	**0.874**	**0.941**	**90.66**	**0.813**	**0.906**	**0.907**
Level 2 10%	kNN	0.902	0.977	93.93	0.879	0.938	0.940
	SVMs	0.932	0.963	97.72	0.894	0.947	0.947
	ET	0.887	0.915	90.12	0.802	0.901	0.901
	Hybrid	**0.925**	**0.973**	**94.87**	**0.897**	**0.948**	**0.949**
Level 3 15%	kNN	0.912	0.978	94.42	0.888	0.943	0.945
	SVMs	0.963	0.977	96.99	0.939	0.970	0.970
	ET	0.893	0.940	91.61	0.832	0.916	0.916
	Hybrid	**0.944**	**0.982**	**96.24**	**0.924**	**0.962**	**0.962**
Level 4 20%	kNN	0.916	0.964	93.98	0.879	0.939	0.940
	SVMs	0.958	0.968	96.31	0.926	0.963	0.963
	ET	0.893	0.943	91.77	0.835	0.917	0.918
	Hybrid	**0.945**	**0.977**	**96.15**	**0.923**	**0.961**	**0.961**
Level 5 25%	kNN	0.862	0.958	90.96	0.819	0.908	0.911
	SVMs	0.912	0.953	93.24	0.864	0.932	0.933
	ET	0.889	0.943	91.58	0.832	0.915	0.916
	Hybrid	**0.910**	**0.971**	**94.04**	**0.881**	**0.940**	**0.941**
Level 6 30%	kNN	0.850	0.952	90.03	0.801	0.898	0.902
	SVMs	0.922	0.942	93.18	0.864	0.932	0.932
	ET	0.889	0.943	91.56	0.831	0.915	0.916
	Hybrid	**0.908**	**0.963**	**93.55**	**0.871**	**0.935**	**0.936**
Level 7 35%	kNN	0.832	0.945	88.72	0.775	0.885	0.888
	SVMs	0.903	0.943	92.32	0.846	0.923	0.924
	ET	0.890	0.944	91.66	0.833	0.916	0.917
	Hybrid	**0.900**	**0.965**	**93.18**	**0.863**	**0.931**	**0.932**
Level 8 40%	kNN	0.817	0.933	87.38	0.748	0.871	0.875
	SVMs	0.905	0.945	92.50	0.850	0.925	0.925
	ET	0.895	0.944	91.90	0.838	0.919	0.919
	Hybrid	**0.901**	**0.963**	**93.16**	**0.863**	**0.931**	**0.932**
Level 9 45%	kNN	0.818	0.939	87.78	0.756	0.875	0.879
	SVMs	0.917	0.948	93.25	0.865	0.932	0.932
	ET	0.885	0.916	90.04	0.801	0.901	901
	Hybrid	**0.907**	**0.959**	**93.27**	**0.865**	**0.932**	**0.933**
Level 10 50%	kNN	0.826	0.937	88.07	0.762	0.878	0.882
	SVMs	0.914	0.948	93.12	0.862	0.931	0.931
	ET	0.882	0.915	89.84	0.797	0.898	0.898
	Hybrid	**0.907**	**0.958**	**93.20**	**0.864**	**0.932**	**0.932**

The results obtained according to the Performance evaluation criteria used for the Feature selection algorithm in PPG signals are shown in Table 10. PPG Feature selection is performed at ten levels. At each level, the model was created by taking 5% of the best features. The amount of features selected for level 1 is 5%, for level 2 is 10%, and for level 5 by increasing, for level 10 is 50%. By using kNN, SVMs, and Ensemble Tree classification algorithms for PPG feature selection and by combining the hybrid artificial intelligence method and classification algorithms, we tried to obtain the best possible results. In the results obtained, the best results were taken at the 8th level, 40%, according to the performance evaluation criteria. An accuracy rate of 0.98 is shown as the closest value to 1. In the tables, the performance value of the model with the best success rate at each level is shown in bold (Tables 9–13).

The results obtained according to the Performance evaluation criteria used for the PPG LF feature selection algorithm are shown in Table 10. The PPG LF feature selection process we performed at ten levels. For each level, kNN, SVMs, and Ensemble Tree classification algorithms were used. By using these algorithms’ optimization methods, it aimed to obtain the best results. By combining the classification algorithms, the best results with the hybrid method were obtained according to the performance evaluation criteria. The best results from these ten levels were obtained at the 7th level; the result was 35%. The accuracy rate displayed is 0.96, with the closest value to 1.

The results obtained according to the performance evaluation criteria used for the PPG MF feature selection algorithm are shown in Table 11. The PPG MF feature selection process we performed at ten levels. For each level, kNN, SVMs, and ensemble tree classification algorithms were used. Using these algorithms and optimization methods was aimed at obtaining the best results. By combining the classification algorithms, the best results with the hybrid method were obtained according to the performance evaluation criteria. The best results from these ten levels were obtained at the 7th level, and the result was 35%. The accuracy rate displayed is 0.97, with the closest value to 1.

The results obtained according to the performance assessment criteria used for the PPG HF feature selection algorithm are shown in Table 12. The PPG HF feature selection process we performed at ten levels. For each level, kNN, SVMs, and Ensemble Tree classification algorithms were used. By using these algorithms’ optimization methods, it aimed to obtain the best results. By combining the classification algorithms, the best results with the hybrid method were obtained according to the performance evaluation criteria. The best results from these ten levels were obtained at the 8th level, the result was 40%. The accuracy rate displayed is 0.98, with the closest value to 1.

The results obtained according to the performance assessment criteria used for the PPG All feature selection algorithm are shown in Table 13. The PPG All feature selection process we performed at ten levels. For each level, kNN, SVMs, and Ensemble Tree classification algorithms were used. By using these algorithms’ optimization methods, it aimed to obtain the best results. By combining the classification algorithms, the best results with the hybrid method were obtained according to the performance evaluation criteria. The best results from these ten levels were obtained at the 3rd level, and the result was 15%. The accuracy rate displayed is 0.98, as the closest value to 1.

Using kNN, SVMs, and Ensemble Tree classification algorithms at each level, the operations are carried out by using optimization at each level. We tried to find the best results using the best performance evaluation criteria. We tried to get the best result for these models by combining the models with the hybrid method.

Twenty-five features were chosen when the produced models were analyzed in terms of signal processing effort before utilizing the feature selection technique. To put it another way, the feature selection method has decreased the feature extraction burden by 70%. The findings acquired based on the signal are shown visually in order to assess the outcomes gained in the research in various ways. The training times of the models are about 30 min, excluding the hybrid model, due to the large data set.

For the reliability of the study, the leave one out method was applied. The obtained training results are summarized in the Table 14.

**Table 14:** Leave-one-out training performance results.

Group	Training					
	Performance evaluation criteria					
	Sensitivity	Specificity	Accuracy rate	Kappa	F-measure	AUC
1	0.98	0.98	99.18	0.97	0.98	0.98
2	0.97	0.97	99.09	0.97	0.98	0.97
3	0.96	0.96	97.29	0.91	0.96	0.96
4	0.97	0.96	98.78	0.98	0.95	0.96
5	0.96	0.95	95.33	0.94	0.93	0.94
6	0.98	0.97	98.2	0.96	0.95	0.97
Average	0.97	0.965	97.98	0.955	0.958	0.963

The hybrid method is a stable classifier formed by weak classifiers. Although its performance is lower in some cases than singular classifiers, it may be preferred more because it is stable and the variance of the answers is low.

## Discussion

The aim of the study is to make the artificial intelligence-based COPD diagnosis quickly and reliably. For this purpose, a different and advanced signal processing and machine learning process from the literature has been developed. More than one model was developed in the study and the comparison of the models is summarized in the results section.

With rapidly polluted air, increased pollution, harsh urban life, and consumption of tobacco products such as tobacco, lung disease is one of today’s most significant health problems (Melekoğlu et al., 2021; Er et al., 2009; Er, Yumusak & Temurtas, 2012). It has been observed that COPD is the most rapidly increasing but least known chest diseases. COPD is a progressive disease that develops due to non-microbial inflammation in the airways due to exposure to cigarette smoke, harmful gases, and particles. As the airways narrow gradually due to this inflammation in the lung tissue, irreversible expansion and destruction occur in the air sacs (alveoli). The changes in chronic obstructive pulmonary disease are irreversible and progressive if the diagnosis is delayed. Therefore, recognition and early diagnosis of this disease are of great importance (Şen et al., 2019). COPD is the third cause of death in the world, and it causes three million deaths annually. With the prevalence of smoking in society, the incidence of COPD and the losses due to this disease are increasing day by day. Therefore, early diagnosis and treatment of this disease are of great importance (Şen et al., 2019).

In a disease where early diagnosis is so important, it has become inevitable to carry out studies for early diagnosis. When we look at the literature, it is seen that the studies on the diagnosis of COPD are generally performed with a stethoscope, demographic information of patients, and spirometers (Şen et al., 2019; Pinto & Marques, 2017; Johari, Balaiyah & Ahmad, 2014). In the literature, the biological signals used for COPD diagnosis are limited to spirometry, stethoscope, and recordings (Şen et al., 2019; Pinto & Marques, 2017; Johari, Balaiyah & Ahmad, 2014). For example, the time required to diagnose COPD with the spirometry device seems to be long and costly. In addition, patients were exposed to many psychological and physiological problems while involved in a long diagnosis process. It has been reported that the level of anxiety in patients increases, and it causes depression in patients (Anar et al., 2012; Akkuş, Karabulutlu & Yağcı, 2016). As diagnosing COPD by spirometry is laborious, complicated, and time-consuming, a minimal number of patients can be included in the process in one day. It has been observed that this causes the prolongation of the diagnosis process of the patients and a decrease in the quality of life of the patient. In addition, as it is an irreversible disease, delayed treatment causes permanent effects on patients.

The use of artificial intelligence algorithms as a decision support system in disease diagnosis is increasing day by day (Joumaa et al., 2022; Char, Abràmoff & Feudtner, 2020; Shailaja, Seetharamulu & Jabbar, 2018). Classical machine learning algorithms are often preferred by researchers because they have low computer capabilities (Ghorbanzadeh et al., 2019; Sahoo, Pradhan & Das, 2020; Zhang et al., 2017). Deep learning approaches work better than classical machine learning algorithms. However, high-capacity computers are required. In this study, a hybrid model is proposed with the help of classical machine learning algorithms close to deep learning performance. In this way, it is aimed to run algorithms on normal computers as well. This approach is quite innovative compared to the literature.

Generally, in the literature, it has been seen that in the studies, more than one single classifier such as DT, SVMs, kNN, multilayer feedforward neural network (MLFFNN), Probabilistic neural network (PNN), and artificial boundary networks are preferred during classification processes (Isik, Guven & Buyukoglan, 2015; Orenc et al., 2017; Örenç, 2019). Ensemble classifier selection is quite rare. In this study, unlike other studies, the Ensemble classifier was used to increase the power of single classifiers, and it was determined that the performance increased significantly. In the literature review, it is thought that using ECG signals of COPD patients will help the diagnosis process of COPD disease to be made quickly and reliably (Uçar et al., 2018b).

In another study for the diagnosis of COPD, with the empirical wavelet transform (EWT) method, kNN, naive Bayes, BayesNet, multilayer perceptron, SVMs, AdaBoost, random subspace, random forest, and J48 algorithm in total nine different types were used and the best performance value for COPD diagnosis was obtained from the classification algorithms in a short time as 5 s (Demir, 2020). In another study, a classification algorithm was used for the diagnosis of COPD. Classification performances are obtained for random forest and J48 decision tree, SVMs, and AdaBoost, respectively, with the ratios of 90.41%, 95.28%, 90.56%, and 85.78%, respectively. It has been demonstrated that the study’s contribution decreases diagnostic time to 5 s while increasing accuracy (Gökçen, 2021). Whereas, in this study, the time has been reduced to 2 s. In this respect, our work is ahead in terms of time. In addition, the performance of the recommended models is 96.96%, better than in the literature. This may be due to the preference for the use of biomedical signals for a reliable signal in the proposed models. Moreover, the detection of sub-frequency bands in signal processing processes is another factor that directly affects model performances.

Studies have shown that PPG signals can help identify COPD. Diagnosis of COPD using PPG signals is said to be achieved in real-time in just 15 s (Ucar et al., 2017). Another research suggests that by utilizing the PPG signal, it may be feasible to diagnose COPD illness in as short as 2 s (Melekoğlu et al., 2021).

All these studies carried out to date contain advantages and disadvantages from each other in terms of different features. This study is an early diagnosis of COPD; in this way, if the treatment process of the patient is started quickly, psychological and physiological difficulties will be minimized.

When the model proposed in this study is compared with the literature, the signal processing process includes innovations in terms of machine learning approach. The use of sub-frequency bands in the signal processing process and the hybrid use of artificial intelligence algorithms are the biggest innovations in the study. Differences in these processes directly affected the performance positively.

## Conclusion

According to the findings of this study, COPD diagnosis might be established by utilizing machine learning with PPG and biological signal processing techniques. It becomes additionally decided that the PPG recording time becomes necessary. With a recording of just 2 s, by using PPG and three sub-frequency signals, three different classification algorithms, combining these models, and using a combined artificial intelligence approach, the exact obtained ratio is 95.31%. These results indicate that the diagnosis of COPD has practical diagnostic methods. The diagnosis of COPD is usually made with a spirometer. However, due to the difficulties of using these devices, different alternatives are needed. The market value of the device is approximately $1,500–$2,500. The proposed model’s rapid diagnosis, reliability, and high accuracy have many advantages because of its technological infrastructure and low cost. The proposed version may be introduced to accessible oxygen saturation gadgets with the usage of easy software. Even with this economic aspect alone, it brings excellent innovation. Therefore, in order to summarize the critical points of the research, it is a system that is (1) easy to use, (2) based on artificial intelligence, (3) has a low economic cost, and (4) makes decisions based on reliable data from biomedical signal measurements. We hope that this research will open new horizons for the diagnosis of COPD.

## Additional Information and Declarations

### Data Availability

The following information was supplied regarding data availability: The data is available at Figshare: Uçar, Muhammed Kürşad (2022): Matlab Data. figshare. Dataset. https://doi.org/10.6084/m9.figshare.21280371.v2. The code is available at Figshare: Uçar, Muhammed Kürşad (2022): Code. figshare. Software. https://doi.org/10.6084/m9.figshare.21280359.v2.
